# Compliance to Iron-Folic Acid Supplementation and Its Association with the Number of ANC Visits in Ethiopia: Systematic Review and Meta-Analysis

**DOI:** 10.1155/2019/3602585

**Published:** 2019-12-22

**Authors:** Yinager Workineh, Ayele Semachew, Emiru Ayalew, Worku Animaw Temesgen

**Affiliations:** ^1^Department of Child Health Nursing, College of Medicine and Health Science, Bahir Dar University, Ethiopia; ^2^Department of Adult Health Nursing, College of Medicine and Health Science, Bahir Dar University, Ethiopia

## Abstract

**Background:**

The World Health Organization recommended that 80% of communities in all countries should receive the standard dose of iron folic acid. But, in Ethiopia, this target was not yet achieved. The compliance of iron folic acid was also variable across each district. Therefore, the aim of this study was to assess women compliance with iron-folic acid supplementation and its association with a number of antenatal care visits in Ethiopia using systematic review and meta-analysis, 2018.

**Methods:**

In the current meta-analysis, the target variables were searched from different electronic database system like PubMed, Google Scholar, Science Direct, and Cochrane Library. To predict the pooled prevalence of compliance with iron-folic acid supplementation in Ethiopia, all original studies were considered. All necessary data were extracted by using a standardized data extraction format. The data were analyzed by using STATA 14 statistical software. Heterogeneity between the studies was assessed by Cochrane *Q* and *I*^2^ tests. A random effect model was computed to estimate the pooled compliance with iron-folic acid supplementation.

**Results:**

Twelve full-text studies were included in the meta-analysis. The findings of this meta-analysis revealed that the pooled prevalence of compliance with iron-folic acid supplementation in Ethiopia was 43.63% (CI: 28.00, 59.25%). The women from the city administration had a high rate of compliance as compared with other regions of Ethiopia. The odds of having four or more antenatal care visit were the independent pooled predictor of compliance with iron-folic acid supplementation.

**Conclusion:**

Current compliance with iron-folic supplementation was lower than the World health organization recommendation. Mothers from the city administration who utilized the antenatal care four and above times, had high level compliance with iron-folic acid supplementation. Therefore, we recommended that women should visit the antenatal clinic four times to compliance with the iron folic acid supplementation.

## 1. Background

Infections, vitamin A, B12, and riboflavin deficiencies as well as blood disorders are the causes for anemia [1]. Globally, iron deficiency anemia is the most common type of anemia which accounted for 50% and 42% of all cases of anemia where among women and children under the age of five respectively [2, 3]. The risk factors of iron deficiency anemia are inadequate dietary intake, malabsorption, high demand during pregnancy, and blood loss due to bleeding.

Deficiencies in iron and folic acid during pregnancy can negatively impact the health of the mother, her pregnancy, as well as fetal development [4]. In this regard, many studies suggested that iron deficiency during pregnancy leads to negative perinatal outcomes, such as low birth weight [5–8], premature birth [6–9], and intrauterine growth retardation [6].

The proportion of iron deficiency anemia (IDA) is varied based on age, sex, and region [10]. The best strategy to prevent anemia related problems is the provision of standard iron and folic acid (IFA) dose at the time of menstruation, pregnancy and adolescent [4, 11]. During pregnancy, oral IFA supplementation is recommended daily in areas where anemia prevalence rate is greater than 20%. On the other hand, weekly provision is indicated when anemia prevalence is at most 20% [11].

The World Health Organization recommended that 80% of communities in all countries should receive the standard dose of iron folic acid. But, from all targeted countries, only Nepal and Senegal have reached greater than 50% for IFA supplementation during pregnancy [12]. Facility and community-based supplementation of IFA to pregnant women was implemented across each region of Ethiopia in order to achieve the WHO recommended level [13]. On the contrary of such effort, coverage of daily iron supplementation has been limited in Ethiopia due to lack of compliance, safety of the drug, and inconsistent availability of drugs at the community level [14–16].

The burden of IDA during pregnancy is not only reduced by initiating of drugs, but also it is highly prevented and controlled by the compliance with IFA supplementation. Compliance, in this case, is defined as taking IFA tablets for 90 days and above [12].

In Ethiopian context, different studies have been conducted to determine the prevalence of compliance with IFA supplementation and associated factors [16–27].

Different studies also revealed that socio-demographic, maternal, and health service related factors were the main determinants of compliance to IFA supplementation. Among the sociodemographic factors of women like age [20, 21, 26], educational status [17, 18, 21, 22, 26], gravidity [26], employment [22], residence [17, 19, 25], and monthly income [22] were significantly associated with compliance to IFA supplementation. Similarly, knowledge of anaemia and iron folate tablets [16, 18, 20–24, 26], number of ANC visit [17, 19, 20, 24], early registration [17, 19, 21, 25, 26], taking number of tablet in each visit [19], taken tablets when sick [22], report of side effects [22], receiving information about the benefit of the tablet [20, 21], counseling on nutritional intake [16, 20, 23, 25], and family support [25] were some of the reproductive services which factors the compliance to IFA supplementation. The last but not the least predictors of compliance to IFA supplementation was the history of anemia [17–19].

As mentioned above, varieties of studies were conducted to estimate the prevalence of IFA compliance in Ethiopia. However, prevalence of IFA compliance ranges from 3.5% [24] to 76% [27] which showed a great variation across different geographical settings and different time periods. The studies conducted before were also with small sample size and their reports were inconsistent and inconclusive. Hence, evidence regarding the exact pooled prevalence of compliance with IFA is required to give an input for public health intervention to anemia in Ethiopia. Therefore, this systematic review and meta-analysis was aimed, firstly, to estimate the pooled prevalence of IFA compliance and secondly, to estimate the effect of the number of ANC on IFA compliance in Ethiopian context.

## 2. Methods

### 2.1. Protocol and Registration

The results of this review were reported based on the Preferred Reporting Items for Systematic Review and Meta-Analysis statement (PRISMA) guideline [28]. There is no registration number.

### 2.2. Eligibility Criteria

All cross-sectional studies were included in the current review. Those studies which had reported the prevalence of IFA compliance and published in English were considered. There was no restriction of the study period. All articles which were available in the search sources from October 29, 2018 to November 29, 2018 were included. Citations without abstract and/or full-text, anonymous reports, editorials, and qualitative studies were excluded from the analysis.

### 2.3. Information Sources

PubMed, Web of Science, Cochrane library, and Google Scholar were accessed. Articles with incomplete reported data were handled through contacting corresponding authors.

### 2.4. Searching Strategy

The core search terms and phrases were “prevalence”, “adherence”, “compliance”, “iron”, “folic acid”, “supplementation”, “utilization”, “uptake”, “number of ANC visit”, and “Ethiopia” were the main key searching terms used to search from October 29, 2018 to November 29, 2018. “OR” or “AND” were used separately and in combination as Boolean operators.

### 2.5. Study Selection

Retrieved studies were exported to reference manager software, Endnote version 7 to remove duplicate studies. Three independent reviewers screened the title and abstract. The disagreement was handled based on established article selection criteria. Three independent authors conducted the abstract and full-text review.

Regarding associated factors, we selected the number of ANC visits to see its effect on the compliance to iron-folic acid supplementation. We selected this factor because of the following reasons: firstly, this factor was the most important factor, which ultimately influence the compliance to iron-folic acid supplementation. Secondly, the effect of the number of ANC visits on compliance to iron-folic acid supplementation has been reported in variable strength of association from different studies [17, 19, 20, 24]. In this regard, the number of ANC visit was strongly and positively associated with compliance to iron-folic acid supplementation in some studies [17, 19, 20]. On the other hand, it was weakly associated with compliance to IFA supplementation in other study [24].

### 2.6. Data Extraction

A standardized data extraction format, which was adopted from the Joanna Briggs Institute (JBI) data extraction format [29], was used to extract data. Three authors (YW, AS, and EA) independently extracted all necessary data using the format. Disagreements at the time of data extraction were resolved through discussion and consensus, and the final consensus was approved by the last (fourth) author (WA). The data extraction format included primary author, publication year, study site, study design, response rate, sample size, prevalence with 95% CI and the quality score of each study.

### 2.7. Outcome Measurement

The outcomes variables of this systematic review are: (a) compliance to IFA supplementation (the primary outcome), which is defined as taking of 90 days and above provided iron-folic acid tablets [12], and (b) the relationship of compliance to IFA supplementation with the number of ANC visits. The prevalence was calculated by dividing the total number of compliance women in all studies reviewed to the total number of pregnant women who involved in the study and multiplying by 100.

(1) $$\begin{array}{c} \text{Pooled IFA compliance}=\left(\frac{\text{Number of compliance}}{\text{number of participants}}\right)*100. \end{array}$$

### 2.8. Quality Assessment

The quality of each cross-sectional study was assessed by using The Newcastle-Ottawa Scale [30]. Three sections of the tool are methodological (graded from five stars focuses on the methodological quality of each study), the comparability of the study and outcomes, and statistical analysis of each original study. The quality of each original study was assessed by three authors independently using this tool. Disagreements between the three authors were resolved by the fourth author. If still there were disagreements between the four authors, the consensus was reached by taking the mean score of the four authors. Finally, researches with a scale of ≥6 out of 10 were considered as high quality.

### 2.9. Statistical Analysis

Publication bias was checked by funnel plot and more objectively through Begg's and Egger's regression test [31]. The heterogeneity of this study was quantified using the *I*-squared statistic, in which 25%, 50%, and 75% represented low, moderate, and high heterogeneity respectively [32, 33]. Pooled analysis was conducted using a weighted inverse variance random-effects model [34]. Subgroup analysis was done on the study region, and the year of publication. Sensitivity analysis was employed to see the effect of single study on the overall estimation. STATA version 14 statistical software was used for meta-analysis.

## 3. Results

### 3.1. Characterstics of Reviewed Studies

**Figure 1:**
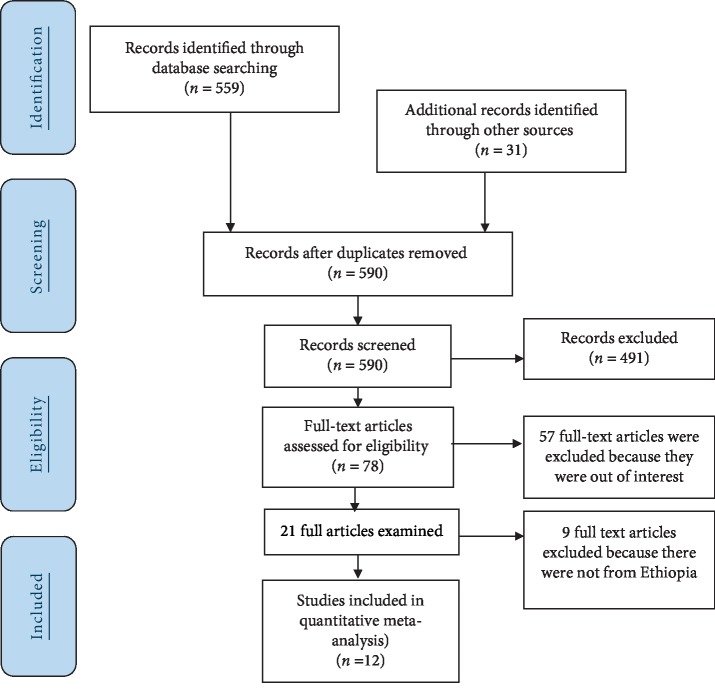
Flowchart to a selection of studies for a systematic review and meta-analysis of the prevalence of compliance to iron-folic acid supplementation and its association with a number of ANC visit in Ethiopia, 2018.

The search strategy retrieved 590 articles from PubMed, Cochrane library, Web of Science, Google Scholar, and other sources. After duplication was removed, 99 articles remained of which 78 were excluded as a result of not fulfilling to our inclusion criteria by reviewing for their titles and abstracts. Then 21 full-text articles were accessed based on inclusion criteria [16, 17, 35–45]. Finally, 12 studies which fulfilled the inclusion criteria [16–27] were included in the meta-analysis (Figure 1).

The eligible twelve studies were published from 2014 to 2018. The total sample size in the current review was 6229. All studies were conducted by using cross-sectional study design. Three of the studies were from the Tigray region [17, 20, 25]; two studies from the Amhara region [18, 19], one from Addis Ababa [22], one from Dire Dawa [27], two from the Oromia region [21, 26], and three from the Southern Nations, Nationalities Peoples' Region [16, 24, 25]. Moreover, five studies were conducted in the community and seven of them form health facilities (Table 1).

### 3.2. Risk Bias Assessment

The Newcastle-Ottawa Scale quality appraisal criteria established for cross-sectional was used. The studies included in this systematic review and meta-analysis had no considerable risk. Therefore, all the studies were considered [16–27] (Table 1).

### 3.3. Meta-Analysis

#### 3.3.1. Prevalence of Compliance to Iron-Folic Acid

**Figure 2:**
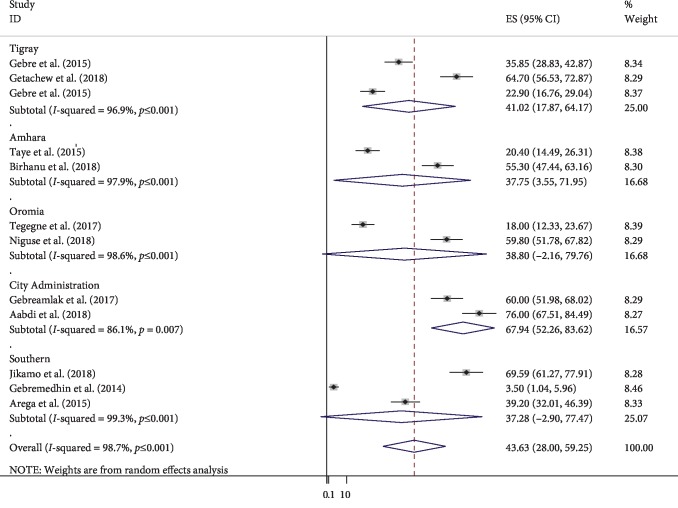
Subgroup prevalence of compliance to iron-folic acid supplementation Ethiopia, 2018 (*n* = 12).

The estimated overall prevalence of compliance to IFA is presented in a forest plot (Figure 2). The overall prevalence of compliance to IFA was 43.63% (95% CI; 28.00, 59.25; *I*^2^ = 98.7%).

### 3.4. Subgroup Analysis

As a result of sever heterogeneity; we performed subgroup analysis based on the geographical setting. Accordingly, the highest prevalence was observed in the city administration (Addis Ababa and Dra Dawa) with prevalence of 67.94% (95% CI: 52.28, 83.62) and the lowest prevalence was observed in southern Ethiopia with prevalence of 37.28% (95% CI: −2.90, 77.47) (Figure 2).

A random effect model was employed to estimate the pooled prevalence of compliance with iron-folic acid supplementation. Different factors associated with the heterogeneity such as publication date, sample size and study site were investigated using univariate meta-regression models. From these variables, none of them were statistically significant (Table 2).

### 3.5. Publication Bias

**Figure 3:**
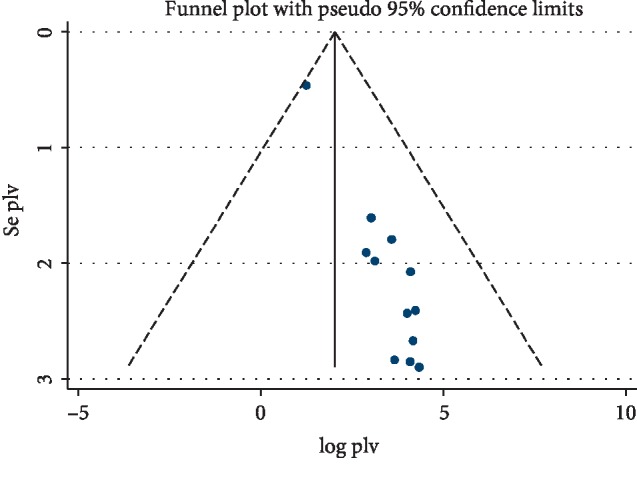
Funnel plot for publication bias, Logprop Or Lnp (Log of Proportion) represented in the X-axis and standard error of log proportion in the Y-axis.

A funnel plot showed asymmetrical distribution (Figure 3). The result of Egger test was also statistically significant for estimating the prevalence of compliance to iron-folic acid supplementation with *Bo* = 1.4 (95% CI 1.2, 1.6) and *p* ≤ 0.001. Trim fill analysis was done to check for further publication bias. In this analysis, there was no difference in the pooled prevalence of compliance to IFA supplementation in this study.

### 3.6. Sensitivity Analysis

**Figure 4:**
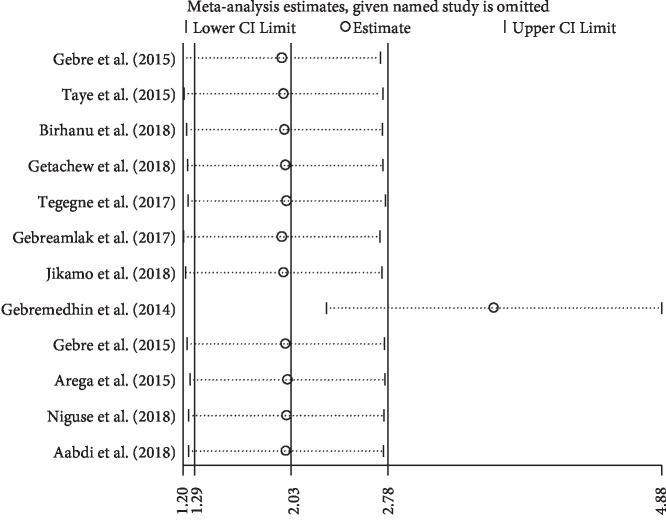
The sensitivity analysis showed the pooled prevalence when the studies omitted step by step.

Among all twelve reviewed studies in the current analysis, the study conducted by Gebremedhin et al. [24] had shown an impact on the overall estimation (Figure 4).

### 3.7. Effect of ANC Visit on Compliance with Iron-Folic Acid Supplementation

**Figure 5:**
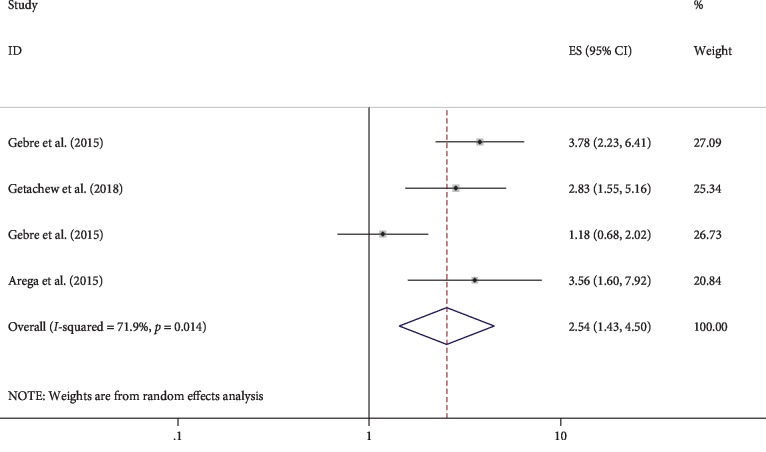
The relationship between the number of ANC visit and compliance with iron-folic acid supplementation in Ethiopia, 2019.

A total of four studies which examined the association between the number of ANC visit and compliance with IFA supplementation were included in the pooled analysis of AOR. Hence, the result in this review indicated the odds of having four or more ANC visits, increases the women compliance to IFA supplementation [OR 2.54 (95% CI: 1.43, 4.50)] (Figure 5).

## 4. Discussion

In this study, systematic review and meta-analysis were used to assess the pooled prevalence of women compliance to IFA supplementation and its association with the number of ANC visit. Although the WHO guidelines recommend that all women should take the IFA dose for at least 90 days and above during their pregnancy time [12], the present meta-analysis reported that only 43.63% (CI: 28.00, 59.25) of women were in compliance with the iron-folic acid supplementation. The prevalence of IFA supplementation is also below the targeted plan of the world health organization.

On the other hand, this finding suggested that the prevalence of compliance with IFA supplementation is much higher than the Ethiopia Demographic and Health survey (EDHS) and other sub Saharan countries [46–55]. The possible explanation for higher rate of compliance with IFA as compared to some sub-Saharan countries could be due to the study site variation in their findings. In the current review, both community and facility level studies were included, but the studies of EDHS were in the community level. The facility level studies in the current study indicated high compliance with the IFA supplementation [22, 23, 27]. The compliance with IFA supplementation in this review is lower than the EDHS results of Ghana [54]. Research indicates that a good majority (96%) of pregnant women in Ghana receive ANC from a trained provider [56].

Based on the subgroup analysis, the prevalence of iron folic acid compliance was high in city administration (Addis Ababa and Dre Dawa) (67.94%) as compared to other regions. Ethiopian demographic and health survey (EDHS) also reported that urban mothers had the high rate of compliance to iron folic acid supplementation as compared to their rural counterparts [46]. In city administrations, there are private clinics and hospitals that give high probability of compliance with IFA supplementations for middle and high income group women. Therefore, women compliance with IFA supplementation would be more in urban settings as compared with the rural counter parts.

Based on the pooled analysis of four AOR of studies, ANC was significantly associated with compliance of IFA. The odds of having four and more ANC was three times higher to the compliance with IFA supplementations. This finding is similar to other studies conducted with EDHS data from 22 countries [47]. The possible justification might be pregnant women who visit ANC service four and more times could acquire a better knowledge of perceived risk and benefit of IFA to prevent anemia during pregnancy.

This study identified ANC visit as an important independent factor for women compliance with IFA supplementation in Ethiopia. National and regional policy and decision makers will have to work on to improve these specific risk factors. Different strategies with appropriate community-based interventions on adherence of ANC visit need to be considered for improving overall compliance of IFA in Ethiopia.

## 5. Limitation

The first limitation of the study was only English articles or reports were considered to carry out this analysis. All studies included in this review were cross-sectional in nature and therefore the outcome variable might be affected by other confounding variables.

## 6. Conclusion

Current compliance with IFA supplementation was lower than the world health organization recommendation. Mothers from city administration who utilized antenatal care for four and above times, had high level compliance with iron-folic acid supplementation. Therefore, health care professionals should focus on increasing compliance with IFA supplementation by providing the recommended number of ANC visits.

## Figures and Tables

**Table 1:** Descriptive summary of 12 studies included in the meta-analysis of prevalence compliance to iron-folic acid supplementation in Ethiopia, 2018.

Region	Study site	Author name	Publication year	Sample size	Response rate	Prevalence	Newcastle-Ottawa Scale
Tigray	Community	Gebre et al.	2015	714	100	35.85	7
	Community	Getachew et al.	2015	320	100	64.70	7
	Facility	Gebre et al.	2015	450	98.4	22.90	7

Amhara	Community	Taye et al.	2015	628	100	20.40	8
	Facility	Birhanu et al.	2018	418	100	55.30	7

Oromia	Facility	Tegegne et al.	2017	405	95.9	18.00	8
	Facility	Niguse et al.	2018	296	93.00	59.80	7

Southern	Facility	Jikamo et al.	2018	365	86.60	69.59	6
	Community	Gebremedhin et al.	2014	1563	97.10	3.50	6
	Community	Arega et al.	2015	296	97.60	39.20	7

City administration	Facility	Gebreamlak et al.	2017	557	88.90	60.00	8
	Facility	Aabdi et al.	2018	217	97.30	76.00	6

**Table 2:** Related factors with heterogeneity of compliance with iron-folic acid supplementation in the Current meta-analysis 2018 (*n* = 12).

Variables	Coefficient	*P* value
Publication years	2.845	0.774
Sample size	−0.018	0.388
Study site at community	−9.50	0.363

## Abbreviations

ANC: Ante natal care
CI: Confidence interval
EDHS: Ethiopia demographic health survey
IFA: Iron-folic acid
WHO: World health organization.
